# DIMPLE: deep insertion, deletion, and missense mutation libraries for exploring protein variation in evolution, disease, and biology

**DOI:** 10.1186/s13059-023-02880-6

**Published:** 2023-02-24

**Authors:** Christian B. Macdonald, David Nedrud, Patrick Rockefeller Grimes, Donovan Trinidad, James S. Fraser, Willow Coyote-Maestas

**Affiliations:** 1 Department of Bioengineering and Therapeutic Sciences, University of California, San Francisco, USA; 2 Bio-Techne, Minneapolis, Minnesota USA; 3 Department of Medicine, Division of Infectious Disease, University of California, San Francisco, USA; 4 Quantitative Biosciences Institute, University of California, San Francisco, USA

## Abstract

Insertions and deletions (indels) enable evolution and cause disease. Due to technical challenges, indels are left out of most mutational scans, limiting our understanding of them in disease, biology, and evolution. We develop a low cost and bias method, DIMPLE, for systematically generating deletions, insertions, and missense mutations in genes, which we test on a range of targets, including Kir2.1. We use DIMPLE to study how indels impact potassium channel structure, disease, and evolution. We find deletions are most disruptive overall, beta sheets are most sensitive to indels, and flexible loops are sensitive to deletions yet tolerate insertions.

**Supplementary Information:**

The online version contains supplementary material available at 10.1186/s13059-023-02880-6.

## Background

Mutations are among the fundamental tools biologists use to understand the nature of genes. To understand how proteins work, biochemists mutate amino acids to learn which are important. Evolutionary biologists reconstruct the history of changes in a gene to understand how that gene’s function changes over time. Synthetic biologists create improved enzymes by introducing mutations and screening for catalytic improvement. Clinical geneticists infer pathogenicity using machine learning that integrates systematic mutational scanning data, conservation patterns, and variant frequencies within patient populations. Each paradigm has produced fundamental insights into how nature produces life and what goes wrong in disease, but each often overlooks mutations beyond simple missense substitutions. Recent work has underscored how essential other types of mutations are to evolutionary novelty and adaptation, as well as their utility for understanding diseases and protein engineering [1–6]. In addition to missense mutations, we must consider frameshifts, recombination, splice variations, and insertions and deletions to evaluate how mutations change proteins. Non-missense mutations present challenges for sequence alignment and evolutionary models and the lack of a biophysical model for how they impact proteins limits their use by protein engineers and understanding by biologists.

Massively parallel mutational scanning, in which mutations are created systematically and then profiled by selection or screening, is commonly used to understand the nature of changes in a protein sequence. Mutational scanning has a long history in experimental biology, starting from pre-molecular techniques such as random cloning for gene mapping [7]. Improved enzymes and sequencing allowed site-directed mutagenesis and iterative small-scale cysteine and alanine scans [8, 9]. These craft-like approaches require iterative mutagenesis and verification for each variant, making them labor-intensive and poorly scale. Error-prone PCR offers simpler access to libraries of mutant sequences, but it is neither programmable nor systematic [10]. The first truly systematic variant libraries were enabled by performing parallel inverse PCR with primers containing degenerate codons (NNN, NNK, etc.), but library composition was constrained by these degenerate codon schemes [11, 12]. Coupling these libraries with sequencing-based phenotypic assays forms the basis for deep mutational scanning (DMS) [13]. DMS studies are enabling fundamental insights in protein biochemistry, evolution, and the molecular bases of disease. These efforts have culminated in large-scale international efforts such as the Atlas of Variant Effects Alliance, with the goal of characterizing all variants circulating within human populations. However, while insertions and deletions (indels) make-up nearly ${1}\!\left/ \!{3}\right.$ of disease-causing variants, to date only two pioneering DMS studies have included indels [1, 6]. Of the two, one contains only single-codon deletions and insertions (but all possible insertions); the other comprehensively samples deletions and single codon insertions, but within a small (129 bp) gene that can be synthesized directly. Neither includes detailed open-source code or molecular biology pipelines, that would enable others to build indel containing libraries. 

DMS studies do not include indels primarily due to technical reasons. Most DMS libraries are constructed using inverse (aka inside-out) PCR, where sequence variation is encoded on one of the priming nucleotides. While inverse PCR works well for missense variant libraries, there are difficulties making deletion or insertions variants. Individual primer pairs would be needed for every variant, and broader T_M_ variations would introduce bias. For this reason, transposons have been used for indel library generation [14–16]. Due to bias intrinsic to transposons, however, these libraries are incomplete, imbalanced, and do not work well for some targets [17, 18]. An alternative to inverse PCR and transposon-based approaches is to leverage microarray-based oligo synthesis (OLS) for making systematic mutational libraries [19–21]. The basic principle of these approaches is to synthesize the variants of interest across all positions of a subregion of a gene and stitch these mutated subregions into a construct by recombination or restriction-ligation cloning. Because each variant is individually synthesized rather than randomly generated, OLS-based libraries are typically more complete, can include any variant type, and simpler to clone than PCR-based approaches. Indeed, the only two mutational scans to date that included indel variants were made with bespoke OLS-based approaches that may not generalize [1, 6].

Here, we present a combined design and experimental pipeline, Deep Indel Missense Programmable Library Engineering (DIMPLE), based on OLS-based synthesis and Golden Gate cloning. DIMPLE is a solution for library design, synthesis, and quality control. Our libraries are an improvement in complexity, completeness, bias, and affordability compared to previous methods. To demonstrate the utility of DIMPLE, we apply it to study how indels impact surface expression of the model potassium channel Kir2.1. This dataset is the first systematic indel scan within a large multi-domain protein, which allows us to empirically explore how insertions and deletions impact protein structure. We compare our data to variants present in the clinic and homologous proteins to explore indels in inward rectifier disease and evolution.

## Results

### A method to generate libraries containing point, insertion, and deletion mutations in parallel

We designed the DIMPLE pipeline to fulfill three major design objectives. First, it should allow for more complex mutations beyond substitutions, in particular multi-codon insertions and deletions. Second, it should produce libraries where there is low bias, and variants are present in roughly equal amounts. Finally, it should be robust and simple to use for experts and non-experts alike. To achieve these objectives, we used a previous library generation pipeline, SPINE, as a scaffold. SPINE was originally developed for domain insertion scanning and later extended to missense mutational scanning [18, 22].

**Fig. 1:**
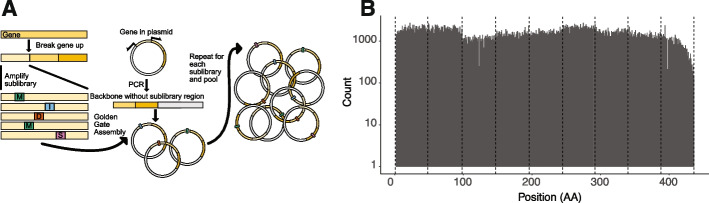
Generation of programmed mutational, deletional, and insertional libraries with DIMPLE in the model potassium channel Kir2.1. **A** Schematic depiction of the library generation process with DIMPLE. **B** Bar plot of mutation type per position against counts. All variants are stacked. Dashed lines indicate the boundaries of each mutagenic sublibrary. Overall, each mutagenized sublibrary region is within 2-fold of each other, indicating well-balanced libraries (Additional file 3: Fig S3B)

DIMPLE is an end-to-end pipeline to allow design, QC, generation, and screening of high-quality, low-bias, indel-containing libraries for most genes of interest. Several technologies and developments have allowed this. First, by encoding mutational diversity in microarray-based oligo pools, we have precise control over exactly which variants are being generated. Second, the use of Golden Gate enzymes allows precise, high-fidelity assembly with no sequence homology requirements. Finally, our computational pipelines are designed to avoid most common problems that can arise during assembly and subpool amplification by choosing specific orthogonal amplification sequences. (Fig 1A). To simplify the process for general users, the software automates the process by generating mutated oligo pools, primers for amplifying sublibraries from the pool, and primers for amplifying each sublibrary’s invariant backbone (https://github.com/odcambc/DIMPLE, Additional file 3: Fig S1) [23]. We are therefore able to removes the bias that occurs with inverse-PCR primer-based and transposon-based libraries and simplify the library creation process at the same time. To assist the community in making DIMPLE libraries, we have prepared a detailed open-source protocol deposited on protocol.io: (10.17504/protocols.io.rm7vzy7k8lx1/v1) [24].

For DIMPLE to be useful to the scientific community at large, it should work on as broad a range of targets as possible. We tested the computational portion of the pipeline against 24 genes ranging in length from 42 to 2561 amino acids and 43% to 59% GC content, yielding 279 sublibrary fragments and 395,330 total variants (Additional file 4: Table S1). In all cases, in silico assembly succeeded in yielding in-frame assemblies with all expected variants present.

We next generated libraries for several of these genes using DIMPLE. In deciding the lengths of insertions and deletions to include in our libraries, we wanted to maximize the potential for insight and specificity while keeping library size manageable. Indel lengths are ultimately constrained by oligo synthesis, with the Agilent 230 bp platform we use allowing for 27 bp deletions and 120 bp insertions. We expect that increasingly long indels would offer diminishing biological insight in most cases, however. The length distribution of deletions in human genomes follows a power law, which suggests that larger deletions are exponentially rare, likely via purifying selection [25]. A prior indel-scanning work provided evidence for this belief, as it revealed that most long indels are deleterious. Departures from this trend were also highly specific to the particular amyloid system, with idiosyncratic effects of large deletions being driven by exposure of a nucleating core and not generalizable to well folded proteins [1]. Based on this, we chose as default lengths 1, 2, and 3 amino acid-long indels, allowing us to capture the most relevant natural variation and observe any interesting length-dependent fitness effects while still maximizing sequencing capacity.

As a demonstration of DIMPLE’s utility, we generated a library with the potassium channel Kir2.1 which contains at every amino acid a mutation to every other amino acid and when possible, a synonymous mutation, 1-3 codon deletions, and 1-3 codon insertions (G, GS, GSG), thus 26 variants per residue. Our insertion sequences were chosen to minimize specific interactions, analogously to GS linkers. We integrated these libraries into stable cell lines using a commonly used high efficiency landing pad cell line method optimized for library generation [26]. DIMPLE is an easy-to-use and customizable computational and experimental pipeline with thorough documentation for generating effective mutational libraries with diverse variant types.

### DIMPLE libraries have even coverage across positions, variant types, and gene targets

Mutational scanning experiments are critically dependent on library quality. In DMS screens, we measure a change in frequency over time, meaning any over- or underrepresented variants in a starting library will decrease assay sensitivity and introduce noise. An ideal library generation method should reliably produce variant pools with even representation (a) across variants at each position, (b) between positions across the target, (c) have nearly all variants present, and (d) be target gene agnostic.

**Fig. 2:**
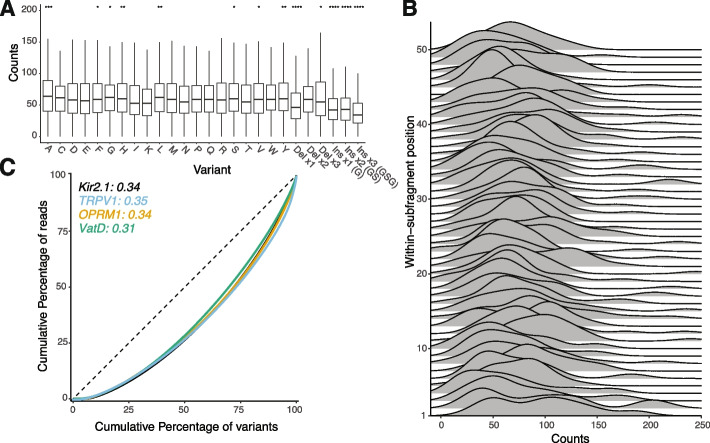
Quantifying the bias of library assembly with DIMPLE. **A** Boxplots of variants at each position across Kir2.1. The vertical length of the box is the interquartile range (IQR), upper bound is the 75th percentile with the lower bound is the 25th percentile. Significance is tested using two-sided *t*-tests controlled for multiple comparisons comparing incorporation means between variants across all positions. Significance levels: ***P < 0.001; **P < 0.01; *P < 0.05, all others not significant. **B** Stacked density plots, or ridge plot ordered bottom-to-top from first to last positions of the second sublibrary of Kir2.1. **C** Lorentz curves and Gini coefficients test the inequality within the distribution of observed variants. A completely even distribution would be a diagonal with a Gini score of 0. The distribution of designed variants for mutagenic libraries of Kir2.1, TRPV1, VatD, and OPRM1 are shown with corresponding Gini scores noted

With DIMPLE, we attempted to simultaneously meet these goals for substitutions, insertions, and deletions. Indels introduce an additional difficulty, as they alter the overall length of a synthesized oligo. In indel libraries, each mutagenic region consists of a range of sizes. We worried this would introduce bias during sublibrary PCR amplification, leading to a systematic bias between variant types. To avoid this bias, for deletion and substitution variants, we included buffer sequences outside the Golden Gate cut sites, adjusted for each variant type, to keep all oligos within a sub-library the same length during amplification but allowing a range of post-assembly sizes (Additional file 3: Fig S2). To test for bias across variant types, we compared the distributions of each variant across the entire gene. We found that most variants are present at similar frequencies; however, in Kir2.1, it appears that indels are present at slightly, but statistically significantly, reduced frequencies. That said, there is less than a two-fold difference between all variant types (Fig. 2A).

Positional bias is another common pathology of DMS libraries and presents a challenge for library generation methods. For microarray-based library generation methods that require a manual sublibrary pooling step, the largest source of positional variability comes from this mixing. This is apparent by eye in the Kir2.1 library, with sublibrary three having the lowest and four having the highest frequency (Fig. 1B). Across Kir2.1, we find that median mutational frequencies across sublibraries are within 2-fold, which indicates evenly represented libraries (Fig. 1B, Additional file 3: Fig S3).

In previous oligo-pool derived libraries, we observed lower variant generation success at the beginnings and ends of sublibraries compared to the middles [18]. To address any positional dependence of digestion or ligation efficiency as a source of bias, DIMPLE includes 4 non-mutated residues from the wildtype sequence flanking the two cut sites. We tested the impact of this modification by comparing the within-sublibrary distributions of variants for each sublibrary (Fig. 2B, Additional file 3: Fig S3). We found no systematic positional biases within sublibraries.

To test the robustness of our technique across different targets from a variety of organisms and classes, we generated additional libraries of a bacterial antibiotic resistance element (VatD from *Enterococcus faecium*), the rat temperature-sensing ion channel TRPV1, and human μ-opioid receptor OPRM1. As with Kir2.1, these libraries contain nearly every variant (VatD-97.5%, TrpV1-97%, and 93.2% out of 5408, 21754, and 10412 possible variants, respectively), with representation at similar frequencies positionally across all sublibraries within twofold of the mean, within twofold by variant types across positions, and similar variant incorporation at positions within sublibraries (Fig 2C, Additional file 3: Fig S4-5). We are thus confident that DIMPLE reliably succeeds at generating missense, insertion, and deletional variants across a range of targets.

In summary, DIMPLE generates libraries that are affordable (<0.30$/variant, Additional file 5: Table S2), near complete, with little bias across positions and variant types, and robust to different targets.

### DIMPLE libraries allow access to unexplored sequence space, revealing how indels impact Kir2.1 surface expression

**Fig. 3:**
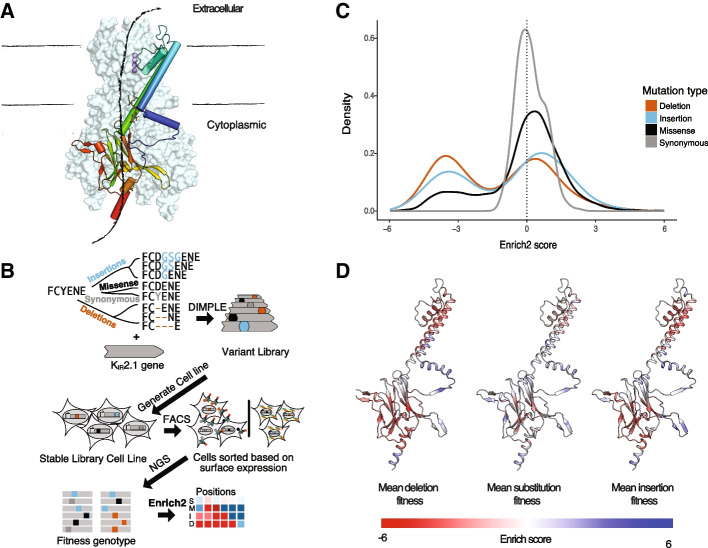
Variable-length indel scanning of Kir2.1 membrane trafficking. **A** Cartoon schematic of Kir architecture: the monomeric structure and overall tetrameric assembly are shown with the crystal structure of Kir2.2 (3SPI). Boundaries of the lipid membrane are indicated with lines, the crystallographic potassium are shown in purple, and locations of the pore highlighted with a cartoon arrow crossing through the channel [29]. **B** Cartoon workflow for studying how different variant types impact Kir2.1 surface expression. Briefly, we use DIMPLE to generate a library including insertion, missense, synonymous, and deletion variants at all positions of Kir2.1, we generate stable HEK293 cell lines, sort these cells based on surface-expression using FACS, perform deep sequencing of these subpopulations, and calculate surface expression fitness scores using Enrich2. **C** The distribution of fitness effects on surface expression of Kir2.1 is displayed as a kernel density estimate. Negative scores indicate decreased trafficking relative to WT Kir2.1. Deletions are the most disruptive perturbation, followed by insertion, missense, and synonymous mutations, respectively. **D**–**F** Mapping the average fitness effects of deletions, substitutions, and insertions across homologous positions in Kir2.2 shows global similarities but local differences between perturbation types. These are plotted from red-white-blue based on surface fitness scores. In general, the structured regions of Kir2.1 are more sensitive to all mutation types

Our initial target, Kir2.1, is a potassium channel with a variety of physiological roles, primarily setting the resting membrane potential of a cell [27]. Many mutations, including deletions, impact Kir2.1 surface expression and cause severe cardiac and developmental disorders [27, 28]. To understand how indels affect Kir2.1 physiology, we performed an assay to identify mutational impacts on surface trafficking. We generated stable cell lines with our Kir2.1 DIMPLE libraries in HEK293T cells, sorted the Kir2.1 DIMPLE libraries based on specific Kir2.1 surface expression with a fluorescent antibody into subpopulations, then sequenced these populations to determine the genotype of variants within each population. By calculating enrichment of variants across surface expression populations relative to WT Kir2.1, we determined that 10964 (out of a total possible 11302, or 97%) variants impact surface expression (Fig 3B, Additional file 3: Fig S6). We used Enrich2 to quantitatively infer the relative impact of variants on surface expression, and we refer to the Enrich2-based scores as “fitness” for simplicity. For these fitness scores, we found high reproducibility between three replicates and our previous study with missense mutations that used the same surface expression assay (Additional file 3: Fig S7).

Across this dataset, we see a clear hierarchy of impacts across mutation types, with (on average) missense mutations being more harmful than synonymous mutations, insertions more pronounced, and most deletions deleterious for trafficking (Fig 3C). The distribution of fitness effects here appears bimodal, with a population of WT-like variants, with a long tail of rare improved-trafficking variants and a second population of poorly trafficked variants. Synonymous mutations preserve the protein sequence, meaning their influences would be limited to second-order effects from translation and/or transcription. As expected, these variants are unimodal and centered around neutrality. Substitutions are extremely context-dependent, with the impact of each depending on the physicochemical context in the structure. Consistent with other indel mutagenesis studies, we observed that deletions were in general more deleterious than insertions [1, 6, 30, 31]. This effect becomes stronger with increasing length for both insertions and deletions as well (Additional file 3: Fig S8).

**Fig. 4:**
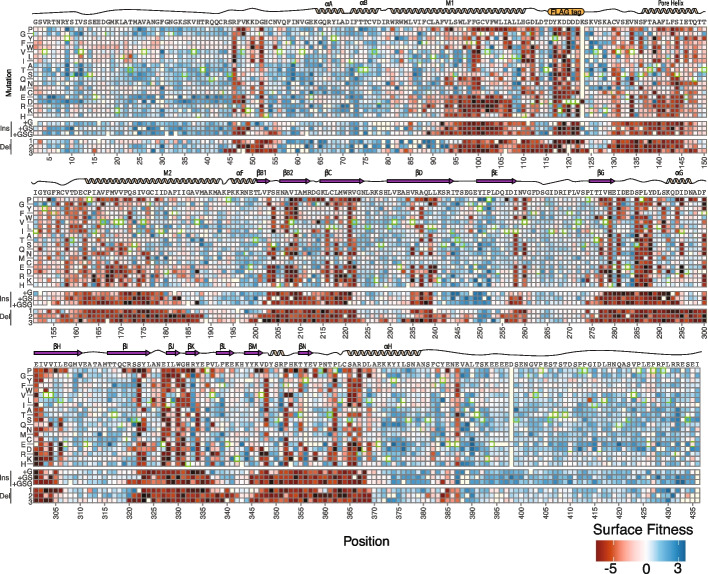
Mutational scanning shows the structural logic of trafficking in Kir2.1. Heatmap of surface expression fitness scores calculated from Enrich2 gradient colored from red (less than WT fitness) to white (WT fitness) to red (greater than WT fitness). Cartoon of secondary structure and labels of structural elements denoted above the heatmap. Only positions for which there were reads in all three replicates are shown here; others were removed in enrichment calculations. Synonymous mutation boxes are outlined with green. Mutations without data are highlighted in light yellow

Examining the pattern of mutational effects on Kir2.1, we found many regions where effects on trafficking were similar across all variant types, and global level variant effects are correlated (Fig. 3D and Fig. 4, Additional file 4: Fig S8-9). As a first attempt at teasing apart the effect of changes to length and physical chemistry, we compared the fitness effects at the same position of inserting a Gly and mutations to Gly. There is a reasonable correlation (Pearson *r* 0.479), but clearly there are distinct effects from each perturbation. In some cases, mechanism underlying disruptive mutations are obvious, such as the FLAG tag (positions 116–123) where mutations disrupt antibody labeling. Across all mutation types, the unstructured N and C termini (positions 1–55 and 378–442) are more mutable than structured regions. Similarly, several flexible loops, such as the βE-βG and βH-βI loops, tolerate any mutations. The helical regions (e.g., H109-L112 and V130-Q147) that determine potassium channel folding as well as folding critical regions of the cytosolic C-terminal domain are completely immutable (e.g., F203-V221, T276-D289, and S322-Y334) [32, 33]. Overall, as in our previous DMS of Kir2.1, structured regions are less mutable than unstructured regions (*p*-value < 2.2e−16 by Wilcoxon rank-sum test, Fig. 3D and Fig. 4, Additional file 3: Fig S10) [34].

Deletions are commonly used by biochemists in an ad hoc fashion to identify important motifs within proteins. For example, Lily Jan’s group identified two motifs within Kir2.1 that were necessary for cell surface expression, the FCYENE (382-386) and SY (322-323) motifs [35]. The FCYENE is a classic example of a diacidic ER-export motif, while the SY motif was later determined to be a Golgi export motif that is a binding interface for the trafficking pathway component AP1 adaptor γ protein [36]. Deletions within the SY motif are extremely deleterious in our assay, while the FCYENE motif deletions are moderately disruptive. The FCYENE is in the distal C terminus in non-folding critical regions meaning mutations here likely solely impact ER-export. In contrast, the SY motif interacts directly with the hydrophobic core so SY variants will additionally suffer dramatic folding deficits [34, 36, 37]. With DIMPLE, we can confirm existing phenotypes within known trafficking motifs and discover new trafficking motifs and their boundaries in less-understood proteins.

### Insertions and deletions have distinct impacts dependent on Kir2.1 secondary structure

**Fig. 5:**
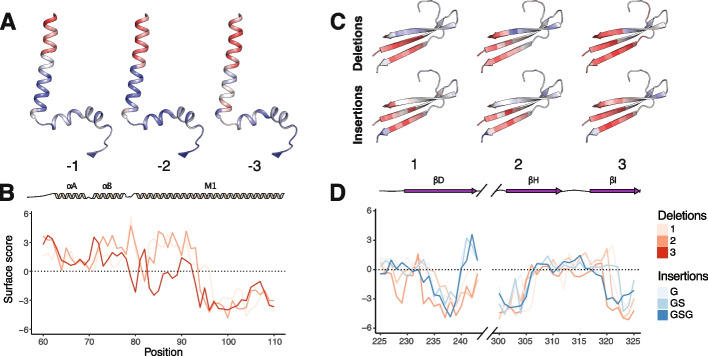
The length of an insertion and deletion impacts Kir2.1 surface expression. **A** Impact of varying the length of deletion on surface expression mapped onto the M1 transmembrane alpha helix and slide helix colored from low-to-high surface expression, red-to-white-to-blue, respectively. **B** Surface scores for the slide-helix position with varying lengths of deletions colored with increasing hue for increasing (or decreasing) length. 1-2 amino acid deletions are tolerated while 3 amino acids result in substantially less surface expression. **C** Impact of varying the length of deletion (top) and insertion (bottom) on surface expression mapped onto three of the immunoglobulin beta sheets colored from low-to-high surface expression, red-to-white-to-blue, respectively. **D** Surface scores for the beta sheet positions, with increasing indel lengths colored with darker hues. The sequences of the segment connecting βD and βH are removed to focus on β sheet

The impact of missense mutations within secondary structural elements depends on the physical chemistry of the mutation. In contrast, indels are broadly disruptive within secondary structural elements and enable a form of secondary structure footprinting. Despite broad similarities across secondary structures, insertions, deletions, and varying lengths distinctly impact Kir2.1 surface expression. For example, within the αA and αB slide helices, deletions are generally beneficial with larger deletions offering the most improvements to surface expression (positive Enrich2 score). This region undergoes a disorder-to-order transition upon ligand binding, perhaps pointing to a tradeoff between folding and function here (Fig. 5A, B). In the M1 helix, by contrast, 1-2 AA length deletions improve surface expression whereas 3 AA deletions and all insertions are neutral or deleterious for surface expression (near-zero or negative Enrich2 score) (Additional file 3: Fig S12). Although it is difficult to know exactly what is happening here, M1’s pivoting in the channel opening could create slack that deletions remove. Due to M1’s role in function, we expect that these variants are non-functional.

To explore how insertions and deletions impact beta sheets, we compared how different lengths of insertions and deletions impact βD, βH, and βI (Fig. 5C, D). βD and βH are for the most part completely intolerant to indels, while βI is surprisingly tolerant to deletions, with the entirety of the beta sheet allowing 1 AA deletions and 2-3 AA deletions allowed in most of the beginning. In βI, G and GS insertions appear to be somewhat tolerated with GSG insertions quite deleterious throughout. While βD and βH are necessary for folding, βI is not. While overall indels within secondary structure elements are disruptive, there are surprising differences in sensitivity between indels with varied lengths within alpha helices and beta sheets. Within Kir2.1, the beta sheets appear far more sensitive to insertions and deletions than alpha helices (*p*-value 0.01201 by Wilcoxon rank sum test, Additional file 3: Fig S13).

### Insertions and deletion in disease and evolution

**Fig. 6:**
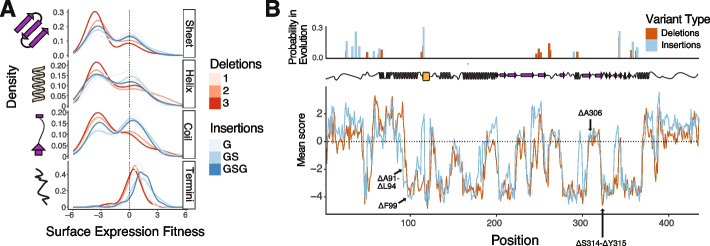
Indels have varying impact in secondary structure, disease, and evolution. **A** The distribution of fitness effects of indels of varying length on surface expression of Kir2.1 divided by secondary structure is displayed as a kernel density estimate. Negative scores indicate decreased trafficking relative to WT Kir2.1. **B** Mean surface scores for deletions and insertions across Kir2.1’s sequence, with conserved insertion and deletion positions in the inward rectifier protein family indicated above, in red and blue as bar plots, respectively. Positions of clinically observed deletions are highlighted with arrows

Insertions and deletions play major roles in disease. On average, ${2}\!\left/ \!{3}\right.$ of these will cause a frame shift and major disruptions. There are also several well-studied examples of in-frame deletions being associated with disorders, including Δ508 in CFTR [38]. There is evidence for the pathogenicity of two deletions in Kir2.1 (ΔA91-L94 and ΔS314-Y315) and two additional deletions are of unknown significance (ΔA306 and ΔF99) [39]. While ΔA91-L94 is not contained within our library because it is four AA long, both ΔA91-ΔA93 and ΔA92-ΔA94 have extremely low surface fitness scores (Fig 6B). Putative pathogenic mutation ΔS314-Y315 and variant of unknown significance (VUS) ΔF99 are both within folding critical regions and have extremely low surface fitness scores and so are likely pathogenic. The VUS ΔA306 is unambiguously neutral in our data despite being in the g-loop, which is critical for potassium conductance. The fitness measured with our assay is surface expression, however; with an additional ion conductance screen, ΔA306 would likely be functionally disruptive and potentially pathogenic. Indel scanning helps us explore the molecular mechanisms and potential pathogenicity of indels in human disease.

Indels occur commonly through errors in DNA replication and recombination [40]. As with all mutations, it is unclear which indels are absent in extant genes due to being too deleterious or if they were never sampled in natural evolution. To compare our experimental results with natural evolution, we determined conserved indel positions in the inward rectifier family by examining the indel states of their Pfam HMM models [41]. This revealed several sites of high-probability insertion and deletion across the sequence, with slightly more deletions vs insertions (15 vs 11, Fig. 6B). Given our observation that deletions are in general more deleterious than insertion, we wondered if there was a pattern to these occurrences. Many of these positions are shared, suggesting that they may be a generally permissive sites towards indels, but a distinct cluster of deletions in βE and the surrounding loops may be a site of specifically deletion-driven diversification. This idea reflects our indel scanning results, which show an improved trafficking phenotype for deletions at these positions. Insertions are allowed which suggests that additional factors may mediate the evolutionary occurrence of indels, including mutational sampling and functional constraints. Certain regions with known functional (but not trafficking) constraints, such as the CD loop, allow deletions in our data which are not observed during evolution [34]. Conversely, a cluster of indels which are extremely deleterious in our data occur within the onset of the H helix, which suggests these positions have become specialized for Kir2.1 trafficking or folding, and perhaps harbor unknown motifs. To attempt to quantify these observations, we stratified the Pfam probabilities into observed or not observed at a site, as they are pseudo-binary. We then calculated the relative occurrence of evolutionary insertions at sites with average positive or negative indel scores. We found that, while there is not a statistically significant relationship for either insertions or deletions, the evidence for association is much stronger for insertions (Fisher’s exact test: *p* = 0.1242 for insertions vs *p* = 0.7732 for deletions). We suspect that a more sophisticated analysis across many different protein families will be necessary to determine the correlation, but overall, we see insertions mostly occurring in permissive positions in our indel scan, while deletions are less tightly coupled.

## Discussion

To summarize, DIMPLE is a robust method that yields high-quality variant libraries with novel multi-codon insertions and deletions in parallel to point mutations and assayed the significance of these for trafficking of a potassium channel Kir2.1. Overall, we observe that insertions and deletions are qualitatively and quantitatively distinct from substitutions with indels more deleterious than missense mutations. By comparing indel fitness across secondary structure, we find that deletions within beta sheets are particularly deleterious while alpha helices have a range of impacts. We find that potential disease-causing deletions are highly deleterious whereas regions with allowed indels during observed inward rectifier diversification also allow indels in our data. Overall, our results highlight the significance of indels for mechanism, disease, and evolution.

Functional genomics approaches such as conventional deep mutational scanning and CRISPRi screens sit on a perturbation continuum. Missense mutations provide information on the physicochemical constraints on a single residue, while CRISPRi provides information on the role of an entire gene within a biological network. Between these two genotypic perturbations there is a noticeable gap in the field. Intermediate perturbations such as motifs, composition, or domains are rarely studied with DMS approaches. Deletional scanning could be useful in identifying which motifs are necessary for membrane protein trafficking. Indel scanning thus fills an important gap in the functional genomics perturbation continuum.

Further work to model how indels influence proteins is clearly necessary, as existing pictures of missense mutations are not sufficient for understanding their impacts [2, 42, 43]. Inserting a sequence might be akin to changing the tension on a spring, with the important parameters being the length and elasticity of the spring. Deletions have the added difficulty of removing sequences entirely, which specifically alters the registry of secondary structural elements and removes interacting residues in addition to increasing the tension on the polymer chain. We suspect an expanded framework will be necessary, considering the physical dynamics of the polymer chain itself in addition to local physicochemical changes as with substitutions. Observational bioinformatic studies of indels across evolution have observed general trends of beta sheets having few indels, alpha helices slightly more indels, and flexible loops and unstructured regions having many indels [2, 42, 43]. As with other less systematic, though still informative, indel scanning studies in structured proteins, we confirm these trends. In contrast to previous indel studies in multi-domain proteins, the truly systematic nature of the data will lend itself for developing empirically based models of how indels alter proteins. Such models would be tremendously useful in understanding the fundamentals of how proteins evolve and how to engineer new proteins.

## Conclusion

Computational and experimental biologists are working to identify how genetic variants impact the function of disease genes. Many of the models for predicting pathogenicity use column-based multiple sequence alignments which typically do not include the gaps that indels cause. Similarly, the mutational scans used for functionally characterizing variants are mostly focused on missense mutations. Overall, this means the impact of indels are undersampled within the ongoing atlas of variant effect. We anticipate that DIMPLE will play a crucial role in filling this gap and enabling the field of mutational scanning to experimentally determine how indels cause disease.

## Methods

### In silico library generation

The DIMPLE software was adapted from SPINE [22] by improving workflow and adding new functions for scanning mutations, insertions, and deletions. Additionally, for ease of use, we added a graphical user interface for those not experienced with command line interface. The first change to the code was incorporating scanning missense mutations which was adapted from a function written for a deep mutational scan (DMS) of a PDZ domain [22]. We improved upon this method by adding the ability to not only mutate each position to the other 19 amino acids but also added the option to mutate to a synonymous codon and a stop codon. These improvements are important for normalization and range in enrichment scores. The other major improvement was to add insertions and deletions at each position. Insertions are defined by the user at the nucleotide level and deletions are defined by the user as the number of nucleotides to delete. Insertions are placed following each amino acid, while deletions delete each amino acid (not including the start codon) and the next consecutive amino acids according to the length specified by the user. Therefore, the deletions stop short of the last amino acid based on the maximum length of deletions. With the addition of insertions and deletions, the size of the oligo changes and therefore needs to be buffered with additional nucleotides to match length for synthesis and for uniform amplification. Additional barcodes were used for buffering the oligo between the primer binding and the type II restriction enzyme recognition site. The size of the buffered region matched the shortest fragment (either largest deletion or smallest insertion) and was uniformly added on the 5′ and 3′ ends. Buffering at this position, however, would disrupt primer binding when using the previous SPINE software since the primer binding sites on the oligo bound partly to the type II restriction enzyme recognition site to maximize the gene fragment size. To remedy this potential issue, the barcode region was expanded so the entire primer could bind. The other changes that were made included fixing the issue of low mutation frequencies at the boundaries of the gene fragments during library generation. To generate more uniform libraries, we added overlap to each fragment by shifting the restriction sites four bases in both directions but did not add mutations in these overlaps to avoid duplication of mutations between fragments. We also added the ability to choose custom codon usage frequencies and fixed an issue with inverse PCR amplification by increasing the melting temperature threshold.

The version of the DIMPLE used in this work has been deposited at https://github.com/odcambc/DIMPLE [23].

All primers designed and used within this manuscript for generating libraries are listed in Additional file 6: Table S3.

### Library generation and cloning

A SurePrint Oligonucleotide library (Agilent Technologies) containing the 58300 oligos for target genes VatD, TRPV1, and OPRM1 was synthesized by Agilent and received as 10 pmol of lyophilized DNA (Additional file 2). This DNA was resuspended in 500 μL 1x TE. Sublibraries were PCR amplified using primer-specific barcodes for each sublibraries and PrimeStar GXL DNA polymerase (Takara Bio) according to the manufacturer’s instructions in 50 μL reactions using 1 μL of the total OLS library as template and 25 cycles of PCR. The reactions were cleaned up using Clean and Concentrate kits (Zymo Research) and eluted in 10 μl of TE buffer. Successful amplification was assessed by running a small amount of the PCR product on an agarose gel.

Vectors containing each gene of interest were synthesized by Twist Bioscience and received as lyophilized plasmid DNA in their High Copy Number Kanamycin backbone and resuspended to 10ng/μL in 1x TE buffer. For Kir2.1, we used the same sequence we had previously used for library generation. For VatD, we designed the library with HindIII and BamHI restriction cut sites for swapping into an expression vector. For OPRM1 and TRPV1, we started with Human and Rat cDNA versions, removed BsmBI and BsaI cut sites using synonymous mutations, and added flanking BsmBI cut sites which cut within CATG and GGGT on the N and C termini of each gene, respectively. These sequences were chosen so that on the N terminus of the gene we encoded for the beginning of the Kozak-start codon and on the C terminus a GS linker.

For each sublibrary, the plasmid was amplified to add on Golden Gate compatible Type IIS restriction sites complementary to those encoded within the sublibrary oligos using Primestar GXL polymerase according to the manufacturer's instructions in 50 μL reactions using 1 μL of the template vector and 25 cycles of PCR. The entire PCR reaction was run on a 0.5% agarose gel and gel purified using a Zymoclean Gel DNA Recovery Kit.

Target gene backbone PCR product and the corresponding oligo sublibrary were assembled using BsaI-mediated Golden Gate cloning. Each 40 μL reaction was composed of 300 ng of backbone DNA, 50 ng of oligo sublibrary DNA, 2 μL BsaI-HF v2 Golden Gate enzyme mixture (New England Biolabs), 4 μL 10x T4 Ligase buffer, and brought up to a total volume of 40 μL with nuclease free water. These reactions were placed in a thermocycler with the following program: (i) 5 min at 37 °C, (ii) 5 min at 16 °C, (iii) repeat (i) and (ii) 29 times, (iv) 5 min at 60 °C, (v) hold at 10 °C. Reactions were cleaned using Zymo Clean and Concentrate kits, eluted into 10μL NFH2O, and transformed into MegaX DH10B (Thermo Fisher) according to manufacturer’s instructions.

Cells were recovered for 1 h at 37 °C. A small subset of the transformed cells were plated at varying cell density to assess transformation efficiency. All transformations had at least 100x the number of transformed colonies compared to the library size. The remaining cell outgrowth was added to 30 mL LB with 50 μg/mL kanamycin and grown at 37 °C with shaking until the OD reached 0.6. Library DNA was isolated by miniprep (Zymo Research). Sublibrary concentration was assessed using Qubit. Each sub-library of a given gene was pooled together at an equimolar ratio. These mixed libraries were assembled with a landing pad cell line compatible backbone containing a Carbenicillin resistance cassette and GSGSGS- P2A-Puromycin cassette for positive selection.

### Sequencing library preparation and genomic DNA extraction and data analysis

Genomic DNA was extracted from sorted cells using a Micro kit from Zymo. Following DNA extraction and quantification with NanoDrop, 1.5 μg of each library was used as template for PCR using cell_line_for_3 and P2A_cell_line_rev primers with PrimeSTAR GXL enzyme, with a final primer concentration of 0.25 μM each, and a T_m_ of 56 °C and 18 cycles. The amplified bands were then run on a 1.5% gel and extracted. The eluted bands were quantified using Qubit with HS kit. For VatD, samples were amplified directly from the miniprepped plasmid library using pGDP3_seq_F and pGDP3_seq_R primers, with an otherwise identical process. For OPRM1 and TrpV1, samples were amplified directly from the miniprepped plasmid library using Landing_pad_backbone_for and P2A_cell_line_rev, using the same methods.

Amplicons were prepared for sequencing using the Nextera XT DNA Library kit from Illumina with 1 ng of DNA input. Samples were indexed using the IDT for Illumina UD indexes and SPRISelect beads at a 0.9x ratio were used for cleanup and final size selection. Each indexed tagmented library was quantified with Qubit HS as well as Agilent 2200 TapeStation. Samples were then pooled and sequenced on a NovaSeq 6000 SP300 flowcell in paired-end mode, generating fastq files for each sample after demultiplexing. Each fastq was then processed in parallel using the following workflow: adapter sequences and contaminants were removed using BBDuk, then paired reads were error corrected with BBMerge and then mapped to the reference sequence using BBMap with 15-mers (all from BBTools [44]). Variants in the mapped SAM file were called using the AnalyzeSaturationMutagenesis tool in GATK v4 [45]. The output of this tool is a csv containing the genotype of each distinct variant as well as the total number of reads. This was then further processed using a python script, which filtered out sequences that were not part of the designed variants, then formatted input files for Enrich2 [46]. Enrichment scores were calculated from the collected processed files using weighted least squares and normalized using wild-type sequences. The final scores were then processed and plotted using R. Read counts are reported within Additional file 8: Table S4 and Enrich2 outputs are in Additional file 2.

Due to the length of synthesized oligos, microarray-based oligo library synthesis (OLS) pools typically have many errors, consisting primarily of single- and multi-base deletions [47, 48]. Analysis of our sequencing results is consistent with this, with most off-target variants observed consisting of large deletions or frameshifts, followed by mismatches (Additional file 4: Fig S11, Additional file 8: Table S5). We observed a consistent trend where assembled products with a truncated mutagenic sublibrary were generated, with an enrichment towards the oligo beginning for larger deletions which makes sense because the oligo is synthesized from 5′-3′ ends. In previous libraries, we observed an error-free final portion of ~15%. In this work, we took advantage of an improved HiFi OLS platform from Agilent, which led to reduced error rates such that 80% of our final Kir2.1 variants consist only of our designed mutations.

The crystal structure of the closely related Kir2.2 was used to model the Kir2.1 structure (PDB: 3SPI). Homologous positions in a sequence alignment were used to map the corresponding position in the Kir2.1 sequence to the structure. An AlphaFold model of mouse Kir2.1 was examined and found to correspond closely to this method but was not used.

For the evolutionary conservation analysis, the central and C-terminal Pfam HMMs (PF01007, PF17655) were downloaded and aligned to Kir2.1. The insertion (or deletion) probability was defined as the probability of transition from a matching to an insertion (or deletion) state at each position in the profile.

### Cell line generation and cell culture

The cells used in this study were engineered by Douglas Fowler’s group and are the 293T LLP-iCasp9 clone 4 strain [26, 34]. These HEK293T landing pad cells were obtained from Douglas Fowler’s group at the University of Washington and authenticated by testing integration of genes in the landing pad backbone with BxB1-comptaible *attB* sites and testing for selection with Blasticidin which was initially used for generating the cell lines. They were tested for mycoplasma before initiating experiments. Prior to transfection, libraries were cloned into a landing pad vector containing a BxB1-compatible a*ttB* recombination site using BsmBI mediated Golden Gate cloning. We kept track of transformation efficiency to maintain library diversity that was at least 100x the size of a given library. We designed the landing pad vector which we recombined the library into to contain BsmBI cut sites with compatible overhangs for the library to have an N terminal Kozak sequence and in-frame with a C-terminal GSGSGS linker-P2A-Puromycin resistance cassette. The Golden Gate protocol we used was 42 °C for 5 min then 16 °C for 10 min repeated for 35 cycles followed by 42 °C for 30 min then 60 °C for 5 min before being stored at 4 °C prior to transfection. This landing pad backbone was generated using Q5 site-directed mutagenesis, according to the manufacturer’s suggestions.

To make the cell lines, 1000 ng of library landing pad constructs were co-transfected with 1000 ng of a BxB1 expression construct (pCAG-NLS-BxB1) using 3.75 μL of lipofectamine 3000 and 5 μL P3000 reagent in 6 wells of a 6 well plate. All cells were cultured in 1X DMEM, 10% FBS, 1% sodium pyruvate, and 1% penicillin/streptomycin (D10). The HEK293T-based cell line has a tetracycline induction cassette upstream of a BxB1 recombination site and split rapamycin analog inducible dimerizable Casp-9. Two days following transfection, expression of integrated genes or iCasp-9 selection system is induced by the addition of doxycycline (2 μg/μL, Sigma-Aldrich) to D10 media. Two days after induction with doxycycline, AP1903 is added (10nM, MedChemExpress) to cause dimerization of Casp9. Successful recombination shifts iCasp-9 out of frame, so only non-recombined cells will die from iCasp-9 induced apoptosis following the addition of AP1903. After 2 days of AP1903-Casp9 selection, the media is changed back to D10 with doxycycline and cells are allowed to recover for 2 days.

Due to the frequent frameshifts or premature stops within OLS-based libraries, we are worried they will introduce noise in our assays. To mitigate this, we typically select for proper in-frame full-length assembly by co-translationally expressing a resistance marker or fluorescent protein downstream of the target gene. This allows facile selection for variants of interest during growth or sorting. In this case, we used puromycin selection. After allowing cells to recover for 2 days, media was changed to D10 with doxycycline and puromycin (2 μg/ml, Life Technologies Corporation), as an additional selection step to remove non-recombined cells. Cells remained in D10 plus doxycycline and puromycin for at least 2 days until cells stopped dying. Following puromycin treatment cells are detached, mixed, and seeded on a 10-cm dish. Cells were then allowed to grow until they reached near confluence, then frozen in aliquots in a cryoprotectant media (90% FBS and 10% DMSO).

### Fluorescence-activated cell sorting

Thawed stocks of library cell lines were seeded on a 10-cm dish in D10 media. The following day, the media was exchanged for fresh D10 to remove cryoprotectant media. Two days prior to the experiment, media was changed to D10 with doxycycline. After 2 days of induction, cells were detached with 1ml TrypLE Express (Thermo Fisher Scientific), pelleted, and washed three times with FACS buffer (5% FBS and 0.1% sodium azide in PBS). Cells were then resuspended in FACS buffer and incubated with a BV-421 anti-DYDDDDK epitope tag antibody (BioLegend) for 1 h at 4 °C. Following incubation with antibody, cells were washed two additional times with FACS buffer before being resuspended at 5 million cells per ml, filtered with cell strainer 5ml tubes (Falcon), covered with aluminum foil, and kept on ice before sorting.

All cell sorting was performed on a BD FACSAria II cell sorter. BV-421 fluorescence was excited with a 405-nm laser and recorded with a 450/50-nm band pass filter. Cells were gated on forward scattering area and side scattering area to separate HEK293T whole cells then forward scattering width and height to find single cells. Surface expressed cells were separated into four subpopulations based on BV-421 fluorescence from the Anti-DYDDDDK antibody. As the library had a clear bimodal distribution, we separated up the gates based on the distribution shapes, such that the first and second gates were of the bottom and upper half of the lower fluorescence populations, while the third and fourth gates were the lower and upper half of the higher fluorescence population. An example gating strategy from the FACSAria Software from the day of a sort is shown in Additional file 3: Fig S6. Cell collected per subpopulation is reported within Additional file 7: Table S4.

## Supplementary Information

**Additional file 1.** Agilent Sure Print OLS Pool. Names and sequences for the OLS pools that we used to build libraries for this study.

**Additional file 2.** Enrich2 outputs for the Kir2.1 surface expression screen. HGVS identifier, surface fitness scores, epsilon, and standard errors for each variant used in this study.

**Additional file 3: Figure S1.** DIMPLE GUI and protocol. **Figure S2.** Design of oligos ordered in oligo library synthesis pools in more detail. **Figure S3.** Kir2.1 Positional coverage. **Figure S4.** Additional library positional coverage. **Figure S5.** Additional library quality measures. **Figure S6.** Gating strategy for sorting Kir2.1 DIMPLE libraries based on surface expression. **Figure S7.** Replicates within and outside of study have high agreement. **Figure S8.** Indel Enrich2 score distributions. **Figure S9.** Distribution of correlations between deletion, insertions, and substitutions within a 10-residue sliding window. **Figure S10.** Kir2.1 standard errors are evenly distributed. **Figure S11.** Score distributions of structured vs. unstructured regions. **Figure S12.** Impact of varying insertion length on M1 and slide helix. **Figure S13.** Score distributions of beta sheets vs. alpha helices across different mutation types. **Figure S14.** Length of deletion errors within Kir2.1 OLS subpools.

**Additional file 4: Table S1.** Inputs and outputs from test running DIMPLE. As discussed within the text, we tested the computational DIMPLE pipeline by including 25 genes. Within this table is their length, GC content, number variants DIMPLE generated, and the number of sub libraries for the gene.

**Additional file 5: Table S2.** Cost for generating DIMPLE libraries. Listed are the reagents used in generating the Kir2.1 library, the number of these reagents used per sublibrary, their estimated cost based on list prices (not-institutionally agreed discounts) as of July 26 2022, the number of reactions for a 9 sublibrary gene 436 amino acids long such as Kir2.1, and total cost for that reagent across the library. Below is listed the total estimated cost for Kir2.1, $3235.34, and cost per variant, $0.28. Both are likely largely over-estimated for academics due to using list prices. Compared to nicking mutagenesis (0.22$/variant in 2016 excluding QC sequencing) is far more expensive than a comparable gene ($0.15/variant) using DIMPLE.

**Additional file 6: Table S3.** Primers used in this study. Primer names and their sequences are listed that were used for amplifying DNA for sequencing and those that were used for generating DIMPLE libraries.

**Additional file 7: Table S4.** Cell Sorting and Sequencing Summary Statistics. Summary statistics for the number of cells collected per subpopulation for Kir2.1, total number of reads for each sample used in this study, number of reads that aligned to variants within the gene, and variants that we filtered for use in figures and fitness calculations because they were expected.

**Additional file 8: Table S5.** Baseline Kir2.1 library errors. Error counts within the sequencing data. Because Illumina sequencing platforms have baseline error rates to a degree this is a combined error between sequencing and OLS DNA synthesis. Errors are broken up between deletions and insertions broken up across multiple length classes, point mutations that would result in synonymous and missense mutations, other which includes multiple mutations within a sequence which is a common within OLS subpool that many oligos with error will have multiple. Also included are the sum total and the number read counts of expected designed variants within the library. From this we can calculate that about 80% of our library are designed variants while 20% are not.

**Additional file 9.** Review history.

## Data Availability

The source code used in this publication is available in the github repository https://github.com/odcambc/DIMPLE/ under the MIT license, and the specific version used in this work has been deposited at Zenodo: https://zenodo.org/record/7574261 [23]. Enrich2 scores are available as Additional file 2. The sequencing data has been deposited at the NCBI Sequence Read Archive as bioproject PRJNA930411 [49]. All code used in processing reads, data analysis, and figure generation has also been deposited in the github repository https://github.com/odcambc/DIMPLE_manuscript_figures.
