# Supplementary material for: DIMPLE: deep insertion, deletion, and missense mutation libraries for exploring protein variation in evolution, disease, and biology
Source: Genome Biol. 2023 Feb 24;24:36. doi: 10.1186/s13059-023-02880-6 (PMC9951526; doi:10.1186/s13059-023-02880-6)
Supplement: Supplementary file 3 — Additional file 3: Figure S1. DIMPLE GUI and protocol. Figure S2. Design of oligos ordered in oligo library synthesis pools in more detail. Figure S3. Kir2.1 Positional coverage. Figure S4. Additional library positional coverage. Figure S5. Additional library quality measures. Figure S6. Gating strategy for sorting Kir2.1 DIMPLE libraries based on surface expression. Figure S7. Replicates within and outside of study have high agreement. Figure S8. Indel Enrich2 score distributions. Figure S9. Distribution of correlations between deletion, insertions, and substitutions within a 10-residue sliding window. Figure S10. Kir2.1 standard errors are evenly distributed. Figure S11. Score distributions of structured vs. unstructured regions. Figure S12. Impact of varying insertion length on M1 and slide helix. Figure S13. Score distributions of beta sheets vs. alpha helices across different mutation types. Figure S14. Length of deletion errors within Kir2.1 OLS subpools. [file 13059_2023_2880_MOESM3_ESM.docx]

Supplemental Figures

**Supplementary figure 1. DIMPLE user friendly GUI and open source protocol. A.** Open source DIMPLE graphical user interface (GUI) and detailed experimental workflow. A. We built a user friendly GUI for DIMPLE that makes library generation easy for those with little computational experience that includes many options for customization. The code is available on Github at: www.github.com/ coywil26/DIMPLE. **B.** Molecular biology can be difficult even when trying to clone a single construct. Cloning is even more challenging when trying to constructs large libraries. We’ve included a detailed step-by-step protocol on bioprotocols with examples that is available at: www.protocols.io/private/ 019898EFCA5611ECBEA10A58A9FEAC02.

**Supplemental Figure 2. Design of oligos ordered in oligo library synthesis pools in more detail. A. Architecture of a designed mutagenic oligo.** Each sublibrary has unique forward and reverse primers for amplification of the sublibrary, spacer sequences that are adjusted to allow for the range of fragments, type IIS restriction sites, which are either BsaI or BsmBI which cut within an unmutated region of the gene of interest and having an additional 4bp of unmutated DNA beyond the cut site to reduce bias in golden gate, and the region of a gene of interest with a single mutation. **B. Variable spacer design to reduce oligo subpool bias.** DIMPLE is designed to include deletions, insertions, and point mutations in parallel within the same subpool. The challenge is that this means there are many different sized fragments which could be amplified at different rates and could result in systematic bias. To reduce the impact of length bias from PCR, we include a variable length spacer sequence (shown in red) which is defined based on the shortest oligo to bring the length equivalent to the longest oligo. To make sure that primer sequences don’t change, the subpool amplifying primer sequence is determined based on the longest oligo then added to the ends of the other oligos within the subpool.

**Supplemental Figure 3. Kir2.1 Positional coverage. A.** Violin plots of every positions across all 9 sublibraries of Kir2.1. These are symmetric density plots that shows the distribution of observed read counts for all variant types at a given position. Overall, we see even representation within each sublibrary for variants, with no systematic bias for any particular region. Any variance between positions is likely more due to difficulties in oligo synthesis or sequencing. There is slightly lower read counts for the end of sublibrary 9 which likely due to tagmentation's well known bias away from the ends of a fragment of DNA. **B.** Boxplots of coverage across Kir2.1 broken apart based on sublibrary. The vertical length of the box is the interquartile range (IQR), upper bound is the 75th percentile with the lower bound is the 25th percentile. Dashed red line indicates mean overall counts per positions across subpools and dotted black lines are 2-fold and 1/2-fold the mean.

**Supplemental Figure 4. A-C.** Barplot of counts of mutation numbers per position for TrpV1, OPRM1, and VatD. The boundaries of each mutagenic sublibrary are indicated with dashed lines. Overall, each mutagenized library is pretty even across subpools, with TrpV1, OPRM1, and VatD all being within x, y, and z fold between medians for a given chunk, respectively. Sequencing libraries were constructed using Nextera which results in poor coverage at the edges which is likely particularly impacting TrpV1 sequencing as the edges of the DNA fragment were closer than ideal. **D-F.** Boxplots of variants at each position across all of TrpV1, OPRM1, and VatD. The vertical length of the box is the interquartile range (IQR), upper bound is the 75th percentile with the lower bound is the 25th percentile. Significance is tested using two-sided T-tests controlled for multiple comparisons comparing incorporation means between variants across all positions. Significance levels: ***P<0.001; **P<0.01; *P<0.05, all others not significant. Across all libraries, different variants are incorporated at similar rates. Sequencing-based counts of designed variant incorporation show small quantitative differences between variants, with different classes being present within x, y, z -fold of each other in TrpV1, OPRM1, and VatD libraries, respectively. Insertions and deletions appear slightly depleted compared to all other variant types in TrpV1. OPRM1 deletions appear to be slightly enriched whereas all other variant types are present at similar frequencies. VatD deletions seem to be slightly enriched as well. Overall, variant types in many different backgrounds are present at similar frequencies. **G- I.** Stacked density plots, or ridge plot ordered bottom-to-top from first to last positions of the second sublibrary of TrpV1, OPRM1, VatD. The second sublibrary was included here because the first and last sub libraries have lower sequencing coverage due to well-known reduced tagmentation efficiency at DNA ends. Overall, we find little positional bias at the beginnings and ends of a sublibrary, which we previously observed in OLS based libraries meaning adding a 4bp unmutated sequence appears to have reduced end based bias.

**Supplemental Figure 5. A-C.** Violin plots of every positions across all 17, 9, and 5 subpools of TrpV1, OPRM1, and VatD genes, respectively. These are symmetric density plots that shows the distribution of observed read counts for all variant types at a given position. Any variance between positions within a subpool is likely more due to difficulties in oligo synthesis or sequencing. Subpool 9 is much smaller than the others, hence why it ends after position 37. There is slightly lower read counts for the end of sublibraries 1 and 17 in TrpV1 and 5 in VatD which are likely due to Nextera tagmentation’s low efficiency at the ends of a fragment of DNA. Overall, we see even representation within each subpool for variants, with no systematic bias for any particular region across subpools. D-F**.** Boxplots of coverage across TrpV1, OPRM1, and VatD broken apart based on sublibrary. The vertical length of the box is the interquartile range (IQR), upper bound is the 75th percentile with the lower bound is the 25th percentile. Dashed red line indicates mean overall counts per positions across subpools and dotted black lines are 2-fold and 1/2-fold the mean. All libraries except for TrpV1 1 and 17 are within two fold of the mean, which is likely due to tagmentation having low efficiencies at the edges of a DNA fragment.

**Supplemental Figure 6. Gating strategy for sorting Kir2.1 DIMPLE libraries based on surface expression. A.** Whole HEK293T cells were gated on forward and side scattering area. **B.** Single cells were gated on forward scattering area and height. CBV-421 fluorescence was excited with a 405nm laser and recorded with a 450/50nm band pass filter. **C.** Cells were then separated into four gates based on increasing BV-421 fluorescence. **D.** Cell counts from FACS experiment in sample of 10,000 events.

**Supplemental Figure 7. Replicates within and outside of study have high agreement. A.** Correlation plots between single replicate fitness scores and fitness distributions for all three replicates. We find good replicability with replicate 1 and 3 most different. As these experiments were done on different days over the course of a week perhaps expression levels were changing. It appears that replicate 1 is less bimodal then replicate 3 which could indicate reduced expression levels and therefore dynamic range. Overall, however we find good agreement between each biological replicate. **B.** As we previously have done a missense mutational scan of Kir2.1, we wanted to test whether we saw agreement. Indeed we see very good agreement between fitness scores. Interestingly, it appears that the overall fitness scores from the experiments are more similar based on a higher Pearson correlation coefficient (0.769 between studied vs 0.762-0.686 between biological replicates). This implies that high repeatability across experiments for the Kir2.1 surface expression screen.

**Supplemental Figure 8. Indel Enrich2 score distributions.** The distribution of fitness effects on surface expression of Kir2.1 from different length insertions and deletions are displayed as kernel density estimates. Negative scores indicate decreased trafficking relative to WT Kir2.1 based on synonymous mutations. As we increase the length of an insertion or deletion, it becomes more deleterious. Overall deletions are more disruptive than insertions.

**Supplemental Figure 9. Distribution of correlations between deletion, insertions, and substitutions within a 10-residue sliding window**. Correlation constants were calculated between each variant type for the 10-residue sliding window centered at every position within the gene. The kernel-smoothed distributions of those correlations are plotted above. At an “average” position, mutational effects are correlated, while at many positions they are uncorrelated or anti-correlated (left tail).

**Supplemental figure 10. Kir2.1 standard error are evenly distributed.** Heatmap of surface expression standard errors scores calculated from Enrich2 gradient colored from white-to-blue . Only positions for which there were reads in all three replicates are shown here; others were removed in enrichment calculations. Synonymous mutation boxes are outlined with green. Overall, standard error is even across the gene and relatively low meaning the data is high quality.

**Supplemental Figure 11. Score distributions of structured vs. unstructured regions.**

**Supplemental figure 12. Impact of varying insertion length on M1 and slide helix. A.** Impact of varying the length of insertion on surface expression mapped onto transmembrane helix M1 and slide helix colored from low- to-neutral-to-high surface expression, red-to-white-to-blue, respectively. **B.** Surface scores for the slide-helix position with varying lengths of insertion colored with increasing hue for increasing length. All insertions are poorly tolerated within the transmembrane likely due to increasing flexibility. Flexible linkers are tolerated within the extracellular loop which intuitively makes sense.

**Supplemental figure 13. Score distributions of beta sheets vs. alpha helices across different mutation types.** The distribution of Enrich2 scores as a function of secondary structure (alpha helix vs beta sheet) and mutation type is plotted.

**Supplemental Figure 14. Length of deletion errors within Kir2.1 OLS subpools. A.** Plotted are the observed length of deletion variants within sublibraries that are not designed within the sublibrary against the position of the codon where it starts. The sublibraries boundaries are indicated by the dashed line. Agilent OLS pools have substantial mutational burden including many deletion variants. Because the oligos are synthesized from 5’-3’ we would expect deletions to start at the 5’ end and decay because it is not possible to have a deletion across sublibrary boundaries. That we see the clean decay implies errors within the libraries are due to oligo synthesis and not our cloning pipeline. Over time as technology improves so will the quality of the libraries. **B-D.** Frequency of errors based of different length non-designed deletions and insertions within the baseline Kir2.1 library. In OLS libraries single base pair deletions are the most common error type which can be clearly seen in **B**. However, frequently there are most frequently multiple errors at once within a mutated oligo that contain single base pair deletions hence the high error count in **D**.
