# Supplementary material for: DIMPLE: deep insertion, deletion, and missense mutation libraries for exploring protein variation in evolution, disease, and biology
Source: Genome Biol. 2023 Feb 24;24:36. doi: 10.1186/s13059-023-02880-6 (PMC9951526; doi:10.1186/s13059-023-02880-6)
Supplement: Supplementary file 9 — Additional file 9. Review history. [file 13059_2023_2880_MOESM9_ESM.docx]

Review History

**First round of review**

**Reviewer 1**

**Were you able to assess all statistics in the manuscript, including the appropriateness of statistical tests used?** There are no statistics in the manuscript.

**Comments to author:**

The manuscript by Macdonald et al. presents a new pipeline (DIMPLE) for the generation of libraries including amino acid insertions, deletions and substitutions in one unique pool. As a proof of concept, the authors also employ DIMPLE to generate an indel library of the potassium channel Kir2.1 and measure how insertions and deletions affect the surface expression of the protein.

The manuscript is sound and I believe the method will be genuinely useful to the deep mutational scanning community as it will promote more studies on comprehensive libraries, speeding up our understanding of the impact of different types of genetic variation on protein function and dysfunction. In its current form - the manuscript starts out as a methodological paper, but then lacks the details required for it to be truly a methodological paper. Instead it then moves on to some biophysical considerations arising from the Kir2.1 data set, yet without validation or without extracting deeper insights from such a dataset.

I think the manuscript is worth publishing, but my advice would be to recalibrate the text in order to strengthen and clarify the methodological part, while also adding some supporting evidence for the conclusions included in the second part.

More specifically:

⁃ p2 Expand the methodological section. A protocol is available on protocols.io, but a straight-forward explanation of the design and experimental steps of Dimple would help the reader a lot, especially if the goal is to engage an audience which may not be familiar with library design or DMS.

⁃ On this same line, figure 1 could be expanded, for example to include what is currently the content of Supplementary Figure 2.

⁃ Again, to make the method accessible and more straightforward, the GitHub documentation and annotation should be improved. Currently, the "Running Test" section uses an example from Spine, their previously published pipeline. It is true Dimple is run in the same way, but it may be confusing for a novel user. In addition, the "user friendly interface" needs to be launched through the command line, but this is not stated anywhere. It took me 30 min to figure this out.

⁃ p1. The authors often mention that their method is cost-efficient and a table reporting costs for this set-up is available in the supplementary materials. Can they compare cost/nt to that of OLS-based approaches?

⁃ Figure 4. Can the author comments on how the impact of mutations on surface expression relates to the impact on protein stability, for example by correlating fitness scores to FoldX deltaGs?

⁃ line 6 p5: the 4 genes targeted are from a variety of organisms and classes. Were they selected on the basis of length?Or sequence complexity? Or any other criteria?

⁃ line 40 p6 'secondary structural elements are less mutable than unstructured regions' : Same as above, this can be exemplified with a simple plot (e.g. vioplot structured vs unstructured regions)

⁃ line 32 p6 "effects on trafficking were similar across variant types". This would benefit from explicit correlation plots (per position) for different variant types. I would add here that a more systematic comparison of mutational impact would be insightful and it seems weird not to do this, once they have this dataset at hand. Can the authors compare insertions to deletions to substitutions and do so - for example - for insertions of and substitutions to the same type of amino acid?

⁃ line 12 p7 'the impact of missense within secondary elements appeared dependent on physicochemistry.. in contrast.. indels are broadly disruptive'. This sentence is a bit misleading as the dataset includes only insertions of G,GS or GSG. Isn't the choice of inserting only G, GS or GSG limiting the insight one can gather from this library? What is the rational behind this choice? The limit of this decision should be stated clearly in the text, or - even better - the authors should include and measure different types of insertions.

⁃ line 21 p8: "insertions cause frameshifts". Frameshifts are not targeted or measured in this study.

⁃ line 30 p8: delta306 would likely be functionally disruptive and pathogenic. Although I agree in principle with this statement, I think a small scale validation of disease variants and a subset of novel variants, would make this point a lot stronger, while also testifying to the robustness of their approach.

⁃ line 12 p9 Probability in evolution should be explicitly correlated with fitness scores

⁃ line 24-26 p9 All of these statements should be supported by corresponding plots to be included in Figure 6.

**Reviewer 2**

**Were you able to assess all statistics in the manuscript, including the appropriateness of statistical tests used?** No, I do not feel adequately qualified to assess the statistics.

**Comments to author:**

The authors, in thorough detail, describe a method for creating libraries of missense, and indels simultaneously. The method is not fundamentally new (see, for example, comment #1 below) but the careful optimization and characterization of the libraries (i.e. designing the process to avoid bias and then extensively characterizing the distribution of resulting mutations) is a strong positive for this paper. The relating of their results with the Kir2.1 protein to structure and known disease variants is also a strong positive. I find it very interesting and surprising the different effects that different length indels can have at some positions.

1. The Church lab made a library based on DNA synthesis and golden gate cloning that included all missense mutations and single codon insertions and deletions (Science 366, 1139-1143 (2019)). The paper is not cited, so I presume the authors are not aware of it. The Church lab used barcoding which the authors do not. How else does the authors' method compare to that of the Church lab?

2. Page 2 line 6 "which may be of benefit for directed evolution efforts". The reader may wonder what this benefit is. Is it because it accesses a diversity that natural mutagenesis is biased to make?

3. P.2 line 8 "but library composition was thus limited to a handful of degenerate codon schemes". Inverse PCR can make any degenerate codon scheme. Can the limitation be more precisely defined?

4. P. 2 line 15. "disease causing genes". I guess this is the key qualifier as other DMS indel studies exist but are not of disease-causing genes (though some of the genes contributes to infectious disease). Why is important to make this distinction in a paper on methodology?

5. P.2 line 18. First sentence. Inverse PCR for missense libraries requires a set of primers for each position. The same is true for indel libraries created by inverse PCR. What is the distinction the authors are trying to make?

6. P.2 line 30. Citation "Seuma et al" is missing the year

7. P.4 lines 18 and 19. The authors seem to be referring to the wrong figure when they twice refer to Figure 1B. What figure did they mean?

8. P.6, bottom of page. It would be good if the authors stated the amino acid positions of the SY and the FCYENE motifs so the reader could easily find them in Figure 4.

9. P. 7 line 16. When the authors use the term "beneficial", do they mean a positive Enrich score or that the Enrich score is statistically higher than that of wildtype (or wildtype synonyms)? I agree with its use if it is the latter, but if it is the former, then I think the authors should just say "positive enrich score."

10. P.8 line 6 "beta sheets overall appear to be far more sensitive to insertions and deletions than alpha helices". The authors could easily perform a statistical test to see if this is true, correct?

11. Last sentence in the legend of Figure 5. I don't understand this sentence.

12. P. 10 line 30. Missing period. "…expression. If we…".

**Authors Response**

**Point-by-point responses to the reviewers’ comments:**

Reviewer 1:

The manuscript by Macdonald et al. presents a new pipeline (DIMPLE) for the generation of libraries including amino acid insertions, deletions and substitutions in one unique pool. As a proof of concept, the authors also employ DIMPLE to generate an indel library of the potassium channel Kir2.1 and measure how insertions and deletions affect the surface expression of the protein.

The manuscript is sound and I believe the method will be genuinely useful to the deep mutational scanning community as it will promote more studies on comprehensive libraries, speeding up our understanding of the impact of different types of genetic variation on protein function and dysfunction. In its current form - the manuscript starts out as a methodological paper, but then lacks the details required for it to be truly a methodological paper. Instead it then moves on to some biophysical considerations arising from the Kir2.1 data set, yet without validation or without extracting deeper insights from such a dataset.

I think the manuscript is worth publishing, but my advice would be to recalibrate the text in order to strengthen and clarify the methodological part, while also adding some supporting evidence for the conclusions included in the second part.

*Response: We appreciate the feedback here, since this is something we struggled with during*

*drafting. While we hope this manuscript serves as an introduction and guide to using*

*DIMPLE, we believe there is something fundamentally different about indel mutational*

*scans, and want to emphasize that our method provides access to new biology via*

*DMS. This means, however, that we are actively trying to understand these*

*perturbations ourselves, and so we feel a full validation of these would expand the*

*scope of this work too much. We have expanded our analyses of our results as well as*

*added additional validation from the literature, however, to allay these valid concerns.*

More specifically:

⁃ p2 Expand the methodological section. A protocol is available on protocols.io, but a straight-forward explanation of the design and experimental steps of Dimple would help the reader a lot, especially if the goal is to engage an audience which may not be familiar with library design or DMS.

⁃ On this same line, figure 1 could be expanded, for example to include what is currently the content of Supplementary Figure 2.

*Response: We have expanded our discussion of the design principles, technologies, and*

*comparative benefits of DIMPLE in the text to address this. We believe that SI figure 2*

*is not necessary for the main text, as we were hoping to display the main workflow at*

*an overview level with figure 1.*

⁃ Again, to make the method accessible and more straightforward, the GitHub documentation and annotation should be improved. Currently, the "Running Test" section uses an example from Spine, their previously published pipeline. It is true Dimple is run in the same way, but it may be confusing for a novel user. In addition, the "user friendly interface" needs to be launched through the command line, but this is not stated anywhere. It took me 30 min to figure this out.

*Response: Thank you for the comments – we have updated the repository to add more*

*documentation and corrected the old reference that was noted.*

⁃ p1. The authors often mention that their method is cost-efficient and a table reporting costs for this set-up is available in the supplementary materials. Can they compare cost/nt to that of OLS-based approaches?

*Response: We’ve updated the SI table to make it comparable to the reported costs from the*

*nicking mutagenesis method of (Wrenbeck et al. 2016), and to allow facile comparison*

*with other methods one may like. These may become stale, though, given how rapidly*

*technology changes and how vendor prices change. We also think that cost/variant is*

*ultimately the appropriate comparison, rather than per nt (that is the approach used in*

*the Wrenbeck manuscript as well).*

⁃ Figure 4. Can the author comments on how the impact of mutations on surface expression relates to the impact on protein stability, for example by correlating fitness scores to FoldX deltaGs?

*Response: We are not sure this would be a useful analysis for several reasons. First, surface*

*expression is not a simple biophysical phenotype: it is the convolution of several*

*simpler properties, of which stability is only one. It would be unclear what a*

*concordance (or discordance) between the two would mean. Secondly, the*

*interpretation of membrane protein in contrast to cytosolic proteins stability in terms of*

*free energy is still developing, and we feel a proper consideration of this would demand*

*a much larger effort that would compose an entirely new project. Finally, some*

*attempts to predict variant effects from similar computational delta Gs have shown less*

*predictive power than purely sequence-based predictors, suggesting that properly*

*incorporating stability calculations is not trivial (Tiemann et al. 2022).*

*We agree that this is an interesting question, however, and have added an additional*

*analysis that hopefully is pertinent. We have determined the correlation between the*

*experimentally-determined Moon and Fleming hydrophobicity scale with observed*

*fitness effects and added this as a new supplementary figure (Moon and Fleming*

*2011). We plan to expand this analysis in follow-up work.*

⁃ line 6 p5: the 4 genes targeted are from a variety of organisms and classes. Were they selected on the basis of length?Or sequence complexity? Or any other criteria?

*Response: We picked these genes to cover a range of length, composition, and type (soluble vs. intrinsic membrane), with a bias towards targets we are interested in. We additionally*

*included several genes that have been studied previously with DMS, including amyloid*

*beta, the target in the only comprehensive indel scanning work to date (Seuma,*

*Lehner, and Bolognesi 2022).*

⁃ line 40 p6 'secondary structural elements are less mutable than unstructured regions' : Same as above, this can be exemplified with a simple plot (e.g. vioplot structured vs unstructured regions)

*Response: Yes, this is a good point – we have provided a new SI figure (8) and a statistical test to quantify this statement.*

⁃ line 32 p6 "effects on trafficking were similar across variant types". This would benefit from explicit correlation plots (per position) for different variant types. I would add here that a more systematic comparison of mutational impact would be insightful and it seems weird not to do this, once they have this dataset at hand. Can the authors compare insertions to deletions to substitutions and do so - for example - for insertions of and substitutions to the same type of amino acid?

*Response: Thank you for another useful suggestion – we have provided a new SI figure (9) and to quantify this (global correlations between deletions, insertion, and substitutions).*

*We also tested the correlation between X->G substitutions and single insertions (i.e.*

*+G) and added the statistical test. While there is a reasonable correlation, we feel the*

*comparison will require some more subtle analysis to properly account for the*

*differences in these types (that is, the length change).*

⁃ line 12 p7 'the impact of missense within secondary elements appeared dependent on physicochemistry.. in contrast.. indels are broadly disruptive'. This sentence is a bit misleading as the dataset includes only insertions of G,GS or GSG. Isn't the choice of inserting only G, GS or GSG limiting the insight one can gather from this library? What is the rational behind this choice? The limit of this decision should be stated clearly in the text, or - even better - the authors should include and measure different types of insertions.

*Response: Thanks for this comment – yes, the rationale was unclear from the text and we have updated it to make this clearer. We chose G, GS, and GSG to isolate the impact of*

*length changes from specific structural or sidechain interactions introduced by the new*

*residues, analogously to a GS linker. We agree that there are interesting experiments*

*to be done with alternative insertion schemes, which we are currently working on. As a*

*generic approach to understand the impact of length changes isolated from specific*

*interactions, however, we believe that a GS-linker-type sequence is appropriate here.*

⁃ line 21 p8: "insertions cause frameshifts". Frameshifts are not targeted or measured in this study.

*Response: Yes, we do not claim to study indels. This sentence is intended to introduce the idea that indels as a class can have properties distinct from nonsynonymous mutations, and*

*that the genotype to phenotype map for indels can be much more complex than for*

*nonsynonymous mutations.*

⁃ line 30 p8: delta306 would likely be functionally disruptive and pathogenic. Although I agree in principle with this statement, I think a small scale validation of disease variants and a subset of novel variants, would make this point a lot stronger, while also testifying to the robustness of their approach.

*Response: We’ve used this approach in our previous manuscripts. Within these we have robustly tested this both based on experiments and previous literature. We found the surface*

*expression screen closely follows literature studies in yeast, mammalian cells, and*

*oocytes. We also measured function for all mutations in this previous study.*

*Furthermore, this antibody site has been used to study surface expression of Kir2.1 for*

*over 20 years. As this manuscript is more of a methods manuscript, we feel it is*

*beyond the scope to do extensive validation experiments especially in light of having*

*done very similar screens in this system previously.*

⁃ line 12 p9 Probability in evolution should be explicitly correlated with fitness scores.

*Response: We have added some additional analysis here. This is slightly subtle, since the*

*insertion probabilities are generated from a protein family, rather than Kir2.1. The*

*probabilities are also nearly binary, so a global comparison might not be appropriate.*

*We plan to quantify this properly across multiple targets in future work, but have added*

*some quantification and statistical tests here.*

⁃ line 24-26 p9 All of these statements should be supported by corresponding plots to be included in Figure 6.

*Response: We have added a statistical test to the manuscript to quantify this.*

Reviewer 2:

The authors, in thorough detail, describe a method for creating libraries of missense, and indels simultaneously. The method is not fundamentally new (see, for example, comment #1 below) but the careful optimization and characterization of the libraries (i.e. designing the process to avoid bias and then extensively characterizing the distribution of resulting mutations) is a strong positive for this paper. The relating of their results with the Kir2.1 protein to structure and known disease variants is also a strong positive. I find it very interesting and surprising the different effects that different length indels can have at some positions.

1. The Church lab made a library based on DNA synthesis and golden gate cloning that included all missense mutations and single codon insertions and deletions (Science 366, 1139-1143 (2019)). The paper is not cited, so I presume the authors are not aware of it. The Church lab used barcoding which the authors do not. How else does the authors' method compare to that of the Church lab?

*Response: This was citation 6 in the original submission. We certainly found this work important and inspiring, but the libraries assayed contained only single deletions. Due to this, any length-dependent effects cannot be determined. Further, their work was not designed to be readily adaptable for other targets, in comparison to DIMPLE.*

*Barcoding is desirable for a variety of reasons. While we did not include it in this work, that is a strength of the Church lab’s approach. Their inclusion of barcodes in their oligo pools necessarily limits the size that sublibraries can span, however, increasing oligo pool costs. It also requires additional cloning steps. We are currently developing approaches to make DIMPLE amenable to barcoding, but that is beyond the scope of this current manuscript.*

2. Page 2 line 6 "which may be of benefit for directed evolution efforts". The reader may wonder what this benefit is. Is it because it accesses a diversity that natural mutagenesis is biased to make?

*Response: We intended to suggest that an unbiased sampling of mutants is not a necessary requirement for directed evolution, rather just a tendency to sample improved variants.*

3. P.2 line 8 "but library composition was thus limited to a handful of degenerate codon schemes". Inverse PCR can make any degenerate codon scheme. Can the limitation be more precisely defined?

*Response: We intended to point out the tradeoffs involved in statistical codon randomization as compared with directly designing mutant sequences. We have expanded the text to clarify.*

4. P. 2 line 15. "disease causing genes". I guess this is the key qualifier as other DMS indel studies exist but are not of disease-causing genes (though some of the genes contributes to infectious disease). Why is important to make this distinction in a paper on methodology?

*Response: We have removed that phrasing since it was extraneous. Our intention here was to point out a gap in the field and underscore the importance of that missing information for understanding disease biology.*

5. P.2 line 18. First sentence. Inverse PCR for missense libraries requires a set of primers for each position. The same is true for indel libraries created by inverse PCR. What is the distinction the authors are trying to make?

*Response: We updated the language to clarify this. We meant to indicate that inverse PCR allows access to all substitutions with a single degenerate primer, while each insertion or deletion would require a new oligo. The cost and input scaling are therefore not ideal. OLS-based approaches scale with cost per variant more directly and thus are better values for these questions.*

6. P.2 line 30. Citation "Seuma et al" is missing the year.

*Response: Fixed.*

7. P.4 lines 18 and 19. The authors seem to be referring to the wrong figure when they twice refer to Figure 1B. What figure did they mean?

*Response: The references to Figure 1B are intentional. The text is discussing whether any apparent uneven variant abundance in our libraries is due to the generation process (and thus inherent to the method) or from inequalities while pooling sublibraries (and thus solvable with more careful pipetting).*

8. P.6, bottom of page. It would be good if the authors stated the amino acid positions of the SY and the FCYENE motifs so the reader could easily find them in Figure 4.

*Response: Yes, this is a good point. We’ve added the position to the text.*

9. P. 7 line 16. When the authors use the term "beneficial", do they mean a positive Enrich score or that the Enrich score is statistically higher than that of wildtype (or wildtype synonyms)? I agree with its use if it is the latter, but if it is the former, then I think the authors should just say "positive enrich score."

*Response: Yes, we are referring to a positive Enrich2 score here. We’ve tweaked the text to make that clear. We also included some additional text clarifying the relationship between the Enrich2 scores and their statistical properties earlier.*

10. P.8 line 6 "beta sheets overall appear to be far more sensitive to insertions and deletions than alpha helices". The authors could easily perform a statistical test to see if this is true, correct?

*Response: Thanks for the suggestion – we agree this would be helpful. We’ve added a test and an SI figure (13) making this comparison.*

11. Last sentence in the legend of Figure 5. I don't understand this sentence.

*Response: We have updated the caption to clarify. We intended to get across that the sheet contains three segments with a loop between that is not displayed in the plot below.*

12. P. 10 line 30. Missing period. "…expression. If we…".

*Response: Fixed.*

**Second round of review**

**Reviewer 1**

It was a pleasure to go through your revised manuscript. Keep up the good work!

**Reviewer 2**

The authors have satisfactorily address all of my comments.
